# Supplementary material for: Plant–Soil Interactions Shape Arbuscular Mycorrhizal Fungal Diversity and Functionality in Eastern Tibetan Meadows
Source: J Fungi (Basel). 2025 Apr 25;11(5):337. doi: 10.3390/jof11050337 (PMC12112861; doi:10.3390/jof11050337)
Supplement: Supplementary file 1 [file jof-11-00337-s001.zip › jof-3531792-supplementary.pdf]

Journal of Fungi

MDPI

Supplementary Materials

PCR amplification

All PCR amplifications were performed on an ABI GeneAMP® 9700 system (ABI, USA), using TransStart Fastpfu DNA polymerase (TransGen Biotech, China). Each PCR run was performed in a 20-μLreaction volume containing 4 μL of 5× FastPtu Buffer, 2 μL of dNTPs (2.5mM), 0.8 μl of 5 μM each primer (5 μM), 0.4 μL of FastPfu polymerase, 0.2 μL of BSA, 10 ng oftemplate DNA, with the remaining volume consisting of sterile distilled water. The PCR cycles went as follows: a denaturation step of 3 minat 95 °C, followed by 30 cycles of denaturation for 20sat 95 °C,annealing for 30 s at 55 °C, and elongation for 45 s at 72 °C, with a final elongation step of 10 min at 72 °C. Every PCR amplification was performed in triplicate, with these three PCR products subsequently pooled together on a per sample basis. AllPCR products werethen purified using the AxyPrep DNA Gel Exaction kit (Axygen Biosciences, USA). This purified set of first PCR products was used in a second PCR step that had 32 cycles (with the other parameters being similar). This (second) set of obtained PCR products were purified once more, using an AxyPrep DNA Gel Exaction kit, after which the final PCR products were pooled on an equimolar concentration basis. The MiSeq library was prepared from the pools of data by using the NEXTflex® Rapid DNA-Seq kit (Bioo Scientific). Finally, each amplicon library was sequenced externally, on an Illumina® MiSeq, by the Shanghai Biozeron Biological Technology Co. Ltd (Shanghai, China), using 2 × 300-bp pair-end sequencing reads.

Table S1. Description of the 49 herbaceous species investigated in the three alpine meadow sites.

Species name	Family name	Functional groups	Life form	Relative abundance of individual species (%)		
				HY	MQ	HZ
Sphallerocarpus gracilis	Apiaceae	Forbs	P	5.56	0	0
Potentilla anserina	Rosaceae	Forbs	P	4.27	3.09	1.58
Galium aparine	Rubiaceae	Forbs	P	10.38	0	0
Allium cyaneum	Amaryllidaceae	Forbs	P	0.94	4.16	0
Euphorbia esula	Euphorbiaceae	Forbs	A	4.62	1.61	0
Polygonum viviparum	Polygonaceae	Forbs	A	2.12	0.47	0
Aster alpinus	Compositae	Forbs	A	4.01	1.96	0
Pedicularis kansuensis	Scrophulariaceae	Forbs	P	0.22	0	0
Lancea tibetica	Mazaceae	Forbs	P	5.55	0.68	2.88
Saussurea hieracioides	Compositae	Forbs	A	1.37	3.25	0
Potentilla bifurca	Rosaceae	Forbs	P	1.67	0	1.62
Anemone rivularis	Ranunculaceae	Forbs	P	2.46	16.16	0.54
Saussurea stella	Compositae	Forbs	P	0.31	3.04	0
Scutellaria hypericifolia	Lamiaceae	Forbs	P	1.356	0	1.78
Leontopodium souliei	Compositae	Forbs	P	2.511	1.54	14.70
Erigeron elongatus	Compositae	Forbs	A	0.063	0	0
Pedicularis chinensis	Orobanchaceae	Forbs	P	3.03	4.10	1.73
Delphinium grandiflorum	Ranunculaceae	Forbs	P	1.979	0	0
Veronica eriogyne	Scrophulariaceae	Forbs	A	0.183	2.26	0
Halenia elliptica	Gentianaceae	Forbs	A	0.851	3.40	0.14
Euphrasia pectinata	Scrophulariaceae	Forbs	A	0.62	0.84	0
Ajuga ciliata	Lamiaceae	Forbs	A	1.648	2.78	0
Ranunculus tanguticus	Ranunculaceae	Forbs	P	0.568	0.29	0
Artemisia stechmanniana	Compositae	Forbs	P	0	1.66	11.47
Plantago major	Plantaginaceae	Forbs	P	0	0.21	0
Taraxacum mongolicum	Compositae	Forbs	A	0	1.76	0.61
Elsholtzia densa	Lamiaceae	Forbs	A	0	2.60	0.52
Biebersteinia heterostemon	Geraniaceae	Forbs	P	0	0.27	0
Cirsium japonicum	Compositae	Forbs	A	0	0.59	0
Saussurea leontodontoides	Compositae	Forbs	P	0	0.67	0
Anaphalis hancockii	Compositae	Forbs	A	0	0.36	0
Picris hieracioides	Compositae	Forbs	A	0	0	1.09
Heteropappus altaicus	Compositae	Forbs	A	0	0	0.57
Galeopsis bifida	Lamiaceae	Forbs	A	0	0	1.28
Salvia roborowskii	Lamiaceae	Forbs	A	0	0	0.64

Fragaria vesca	Rosaceae	Forbs	P	0	0	1.17
Cumulative relative abundance for functional group of forbs				56.27	57.73	42.31
Gueldenstaedtia verna	Leguminosae	Legumes	P	1.51	2.42	1.17
Vicia sepium	Leguminosae	Legumes	A	0.73	0	1.17
Astragalus polycladus	Leguminosae	Legumes	P	0.50	1.19	0.424
Thermopsis lanceolata	Leguminosae	Legumes	P	0	3.32	0
Medicago ruthenica	Leguminosae	Legumes	A	0	0.473	4.383
Cumulative relative abundance for functional group of legumes				2.74	7.40	7.15
Kobresia pygmaea	Cyperaceae	Sedges	P	16.12	0	1.88
Kobresia graminifolia	Cyperaceae	Sedges	P	0	16.80	1.75
Elymus nutans	Gramineae	Grasses	A	1.84	0.62	28.79
Poa pratensis	Gramineae	Grasses	A	6.565	3.96	2.90
Calamagrostis epigeios	Gramineae	Grasses	A	2.225	4.07	7.94
Stipa aliena	Gramineae	Grasses	P	8.053	1.41	0
Agrostis hugoniana	Gramineae	Grasses	P	0	2.35	0
Leymus secalinus	Gramineae	Grasses	P	0	0	1.43
Cumulative relative abundance for functional group of grassess				34.80	29.21	44.68

A: Annual, P: perennial. Each species had more than 10 plantsin each sampling plot. Hongyuan (HY), Maqu (MQ), Hezuo (HZ).

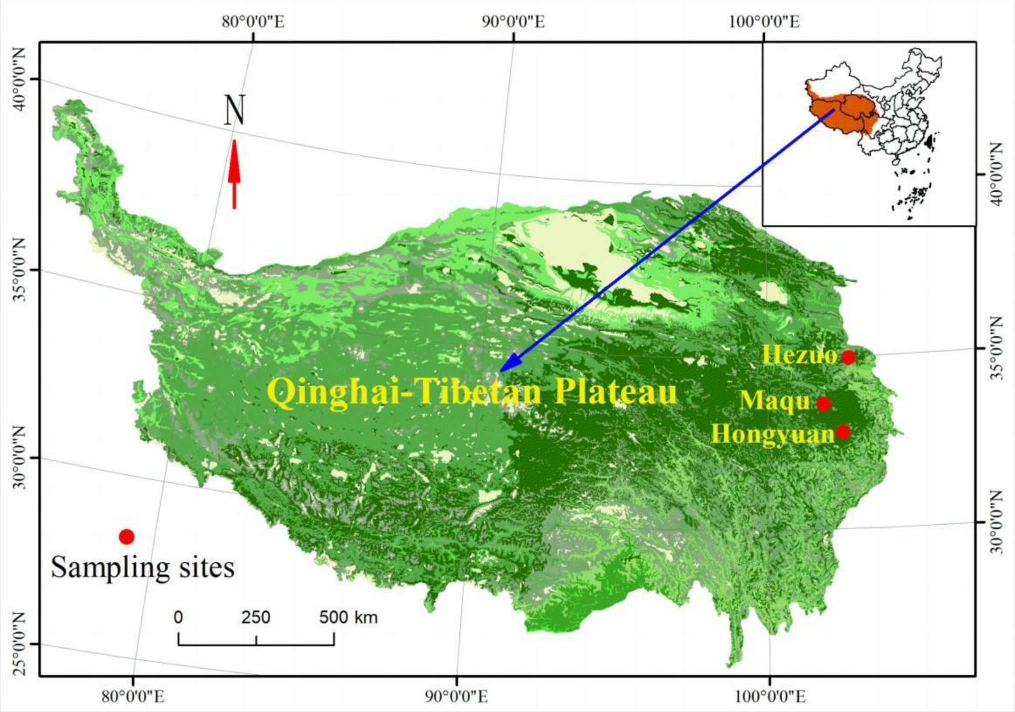

Figure S1. Map showing the location of the three alpine meadow sites (Hongyuan, Maqu, and Hezuo) sampled on the eastern of Tibetan Plateau, China.

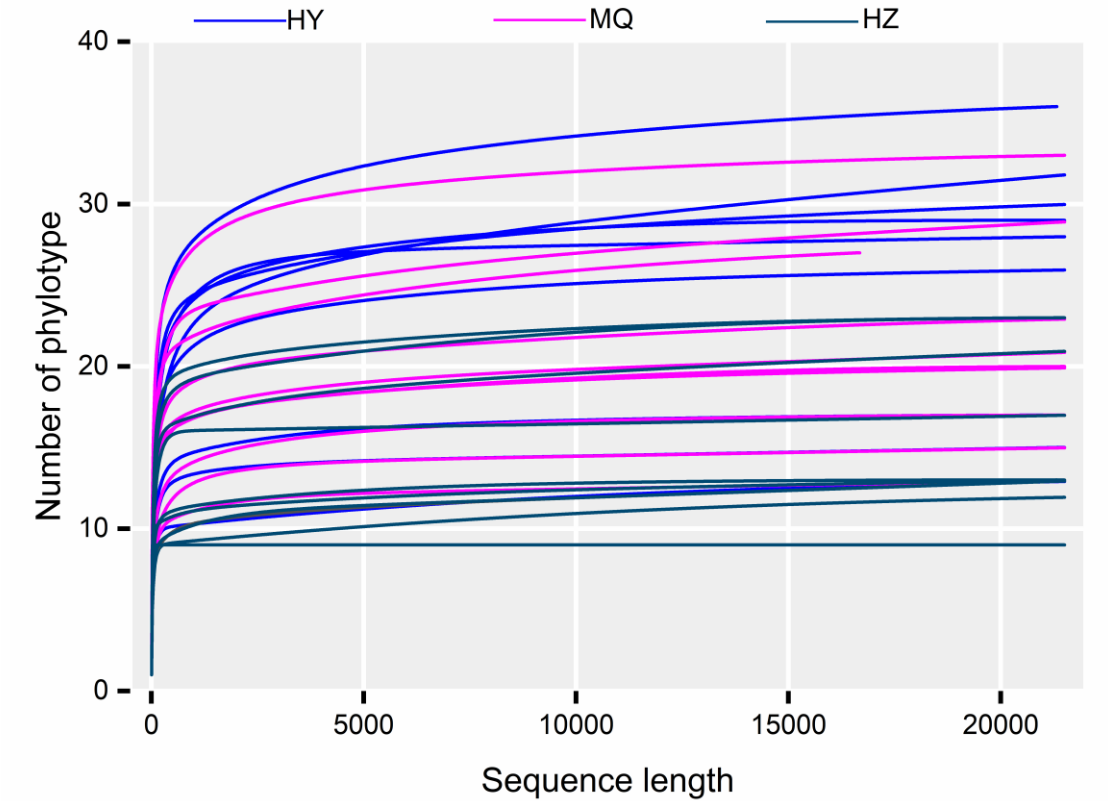

Figure S2. Rarefaction curves showing the AM fungal phylotype abundances across all samples in the three alpine meadow sites of this study.
